# Pyrrolidine Dithiocarbamate Attenuates Cardiocyte Apoptosis and Ameliorates Heart Failure Following Coronary Microembolization in Rats

**DOI:** 10.4274/balkanmedj.galenos.2019.2019.3.8

**Published:** 2019-07-11

**Authors:** Shumei Li, Jun Fang, Lianglong Chen

**Affiliations:** 1 Department of Cardiology, Union Hospital, Fujian Medical University, Fujian, China

**Keywords:** Cardiac function, cardiocyte apoptosis, nuclear factor-kB, pyrrolidine dithiocarbamate

## Abstract

**Background:**

Nuclear factor-kB is highly activated in cardiovascular disorders. However, few articles have targeted at the role of nuclear factor-kB inhibitor in heart failure.

**Aims:**

To evaluate the effects of nuclear factor-kB inhibitor pyrrolidine dithiocarbamate on cardiocyte apoptosis and cardiac function in a rat heart failure model.

**Study Design:**

Animal experiment.

**Methods:**

A stable and reproducible rat heart failure model (n=64) was prepared by injecting homologous microthrombotic particles into the left ventricle of Sprague–Dawley rats while obstructing the ascending aorta to produce coronary microembolization. Rats with heart failure were randomized into untreated (HFu) and pyrrolidine dithiocarbamate-treated (HFp) groups; the latter received an intraperitoneal injection of pyrrolidine dithiocarbamate (100 mg/kg/day) 1 h prior to surgery as well as on postoperative days 1-7. The sham group comprised 32 Sprague–Dawley rats. Eight rats from each group were sacrificed on days 1, 3, 7, and 14 postoperatively. Masson’s trichrome staining was used to determine the micro-fibrotic area to indicate the severity of myocardial loss. Terminal transferase uridine triphosphate nick end labeling staining was used to detect apoptotic cardiomyocytes. Echocardiography and hemodynamics were performed to evaluate left ventricular function.

**Results:**

Rats with heart failure exhibited pathological changes evidenced by patchy myocardial fibrosis, remarkably elevated severity of myocardial loss, and persistently reduced left ventricular function. At the end of the study, compared with the HFu group, myocardial infarct size was reduced by 28% (p=0.001), cardiocyte apoptosis was suppressed (7.17%±1.47% vs 2.83%±0.75%, p<0.001), cardiac function parameters such as left ventricular ejection fraction (80%±4% vs 61%±6%), left ventricular + dP/dt max (4828±289 vs 2918±76 mmHg.s−1), left ventricular - dP/dt max (4398±269 vs 2481±365 mmHg.s−1), and left ventricular systolic pressure (126±13 vs 100±10 mmHg) were significantly increased, and left ventricular end-diastolic pressure was reduced (18±2 vs 13±1 mmHg) (p<0.001, for all) in the HFu group.

**Conclusion:**

Our rat model can adequately mimic heart failure via coronary vessel embolization. Moreover, pyrrolidine dithiocarbamate treatment can reduce cardiocyte apoptosis and improve cardiac function, which may be beneficial for patients with heart failure secondary to myocardial infarction.

Congestive heart failure (HF) is a serious and complex clinical condition with high morbidity, disability rate, and mortality. Patients with coronary heart disease, particularly those receiving percutaneous coronary intervention, can easily become prone to HF due to coronary microembolization (1). Therefore, the current study created a reproducible and stable rat chronic HF model using homologous blood emboli to produce coronary microembolization. This paper outlines the technique and characterizes its hemodynamic effects in rats.

The available literature on cardiovascular disorders has documented that nuclear factor-kB (NF-kB) is highly activated (2,3,4). NF-kB can enhance the inflammatory response in patients with unstable angina pectoris with elevated C-reactive protein levels (3). In addition, it can be selectively and markedly activated in peripheral blood leukocytes of people with unstable angina before malignant cardiac events by mechanistically engaging in plaque disruption that can induce acute coronary artery syndromes (5). Recent studies have reported that NF-kB mediates autophagy induction to aggravate myocardial injury in cardiac ischemia/reperfusion injury and post-myocardial infarction cardiac remodeling (6). Furthermore, it may cause cardiac hypertrophy and hypertension by regulating cytokines and oxidative stress (7). More evidence has shown that NF-kB regulates the inflammatory cascade and apoptosis-associated genes, which are a confirmed cause for progressive contractile dysfunction and cardiac injury (8). A previous study had reported an attenuated cardiac function after myocardial infarction in NF-κB-null mice (9). Oxidative stress can activate NF-κB and trigger the transcription of pro-apoptotic genes, such as Fas, Bax, and FasL, resulting in myocardial cell apoptosis and HF. A study has documented that pyrrolidine dithiocarbamate (PDTC), an antioxidant, specifically suppresses NF-kB activation (10). However, few articles have explored the role of this NF-kB inhibitor in HF. Therefore, the present study attempts to investigate whether PDTC-induced NF-kB inhibition can reduce cardiac injury and improve progression of contractile dysfunction in rats with HF.

## MATERIALS AND METHODS

### Animal preparation and experimental procedures

Sprague Dawley (SD) rats (weighing 280-320 g) were provided from the animal center of Medical University. These animals were raised in conditions with controlled humidity, lighting cycle, and temperature and were allowed *ad libitum* access to water and rat chow. The study protocol observed *The Guide for the Care and Use of Laboratory Animals* (NIH publication no: 85-23, revised 1996) and received approval from the Clinical and Animal Research Ethics Committee of Medical University.

### Preparation of homologous microthrombotic particles

Male Sprague Dawley rats were sedated by intraperitoneal injection of ketamine (75 mg/kg) and diazepam (7.5 mg/kg) 36 h before the experiment. Prior to each injection, 7 mg of the particles (~15-19x10^9^) were thoroughly suspended in calcium-free Dulbecco’s phosphate-buffered saline with a vortex agitator. Tail blood (500 µL per rat) was obtained and stored at room temperature to allow clot formation. The clot was then fragmented with a homogenizer and filtered using a 38 µm screen to obtain microthrombi (microthrombi-emboli), which were between 10-30 µm as measured by a micrometer.

### Creation of HF model

After 36 h, the rats were reanaesthetized with ether and placed on a small animal ventilator. Next, a thoracotomy along the midline was conducted with the mammalian arteries ligated. Afterwards, a sternotomy was performed between the second and third intercostal spaces. The pericardium was cut open, exposing the ascending aorta. A bolus of the microembolic suspension in phosphate-buffered saline was injected into the left ventricular (LV) chamber with a 26-gage needle while the ascending aorta was occluded for 10 s. Sutures were used to close the thoracic cavity and skin incision. The age-matched sham group received the same surgical procedure and a phosphate-buffered saline injection of the same volume.

### Experimental protocol

A total of 32 Sprague Dawley rats received a sham operation and 64 rats underwent the previously described surgery to establish an HF model. Rats with HF were randomized into an untreated (HFu, n=32) and PDTC-treated (HFp, n=32) groups. The rats with HF were sacrificed at postoperative days 1, 3, 7, and 14, forming four subgroups (n=8 each). PDTC (Sigma, USA) in a 0.9% NaCl solution was intraperitoneally administrated at a dosage of 100 mg/kg/day, starting 1 h prior to surgery and continuing for 7 days postoperatively. The PDTC doses have been proved to effectively blunt NF-kB activation with no significant toxicity (11).

### Study protocols

### Echocardiographic study

The rats were sedated for the echocardiography procedure, as previously described. Echocardiography with two-dimensional and M-mode imaging modalities was conducted with a medical diagnostic system (VIVD-7, GE Medical System, USA) equipped with a 10 mHz phased-array transducer. Electrocardiographic measurements were simultaneously recorded with respiratory tracings. The LV end-diastolic diameter (left ventricular end diastolic dia- meter; at the R wave of the electrocardiography) and the end-systolic diameter (LV end-systolic dimension; at the peak inward motion of the endocardial excursion) were recorded using the M-mode tracings of the level of the chordae tendinae at the end of expiration in the LV short axis view. A cubic formula was employed to calculate the LV ejection fraction. For all parameters, at least three consecutive cardiac cycles were averaged. The echocardiographic readings were recorded and stored throughout the experiment.

### Hemodynamic study

Hemodynamic measurements were performed under general sedation and a 20-gage polyethylene cannula was inserted from the right carotid artery into the left ventricle. The maximum change of LV pressure during isovolumic contraction (LV + dP/dt max) and relaxation (LV - dP/dt max) was calculated from the LV pressure traced with a resistance-capacitance analog differentiator and the LV systolic pressure. The hemodynamic and functional parameters were recorded in each procedure.

### Histopathological study

After the harvest, the hearts were fixed in 10% formalin, embedded and cut into slices (5 µm thick). Trans-sections from the chordae tendineae, papillary muscle, mitral valve, and near-apex of the LV were subject to morphological assessment.

Hematoxylin and eosin staining was performed on the sections to evaluate the general morphology. Masson’s trichrome staining showed micro-fibrotic foci at the chronic phases (14 postoperative days), indicating myocardial loss. The area taken by fibrotic foci, representing the severity of myocardial loss, was calculated with the following equation: severity of myocardial loss (%): total fibrotic area/total area of a whole section (×100). The whole section was morphologically quantified using an image processing system (Image-Pro Plus 5.0 Media Cybernetics, Inc, USA).

### Detection of apoptosis by TUNEL assay

Myocardial tissues were placed in a paraffin block and transversely cut into sections (5 μm thick) based on the instructions of an *in situ* cell death detection kit (Roche, Switzerland). The apoptosis of cardiomyocytes was measured by the terminal transferase uridine triphosphate nick end labeling (TUNEL) staining. The treated myocardial tissue was heated in a sodium citrate solution and dissolved with proteinase K for DNA exposure. The resulting DNA strand breaks were labeled with a terminal transferase enzyme, with dUTP molecules conjugated to horseradish peroxidase, and visualized by immunohistochemistry. TUNEL-positive nuclei of cardiomyocytes (brownish red) were regarded as apoptotic cells from 10 random fields (≈400) per section with a total of 100 nuclei per field. The severity of apoptosis was evaluated by the apoptosis index, calculated as the number of TUNEL-positive nuclei over the total nuclei.

### Statistical analysis

Data was expressed as mean ± standard deviation. SPSS 13.0 for Windows was used to analyze the data. Intergroup comparisons were performed by one-way ANOVA followed by Dunnett’s modified test. Statistical significance was set at p<0.05.

## RESULTS

### General conditions of experimental rats

All the animals in the sham group survived the operation. Six rats in the HFu group died within 1 week after coronary microembolization and the survivors showed various degrees of mental exhaustion, dyspnea, decreased activity and eating, and shock in severe cases. Autopsy of dead rats showed bilateral pulmonary congestion and pleural effusion, suggesting sudden cardiac death and possible death from HF. Two rats died in the HFp group.

### Histopathological findings

**Figure 1:**
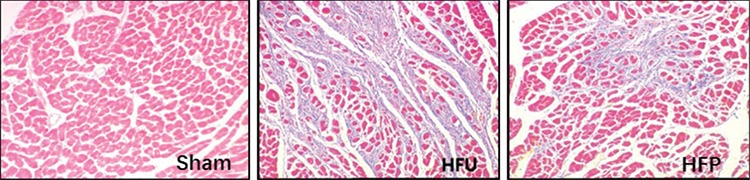
PDTC administration significantly suppressed myocardial fibrosis following coronary microembolization. PDTC: pyrrolidine dithiocarbamate

After Masson’s trichrome staining, qualitative analysis revealed multiple patchy fibrosis in the HF group on postoperative day 14. Heterogeneous distribution of transmural changes showed more involvement of the sub-endocardial rather than the sub-epicardial region (Figure 1). Compared with the sham group, severity of myocardial loss was significantly increased in CME, (21.25%±3.54% vs 0.66%±0.35%; p<0.001; Table 1). PDTC administration significantly reduced the myocardial infarct size in the HFp group. On postoperative day 14, severity of myocardial loss markedly decreased in the HFp group compared to the HFu group (by 28%, p=0.001).

### Effects of PDTC on cardiomyocyte apoptosis

**Figure 2:**
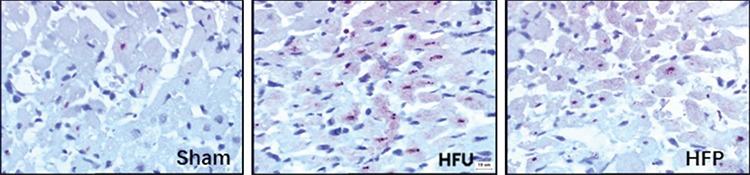
PDTC treatment significantly reduced myocardial apoptosis rate compared with that of the HFu group. PDTC: pyrrolidine dithiocarbamate

The death of cardiac muscle cells occurs in two forms: necrosis and apoptosis. Compared to the sham group, apoptosis was higher in the myocardial microinfarct zone of the HF group (Figure 2). On postoperative day 1, 3, 7, and 14, cardiomyocyte apoptosis indices were 13.33%±2.16%, 19.67%±2.58%, 11.50%±2.74%, and 7.17%±1.47% (p<0.001 for all). PDTC treatment significantly reduced myocardial apoptosis rate (Table 2).

### Alterations in cardiac function

Compared to their sham counterparts, the HFu rats had markedly lower left ventricular systolic pressure, LV + dP/dt max, LV - dP/dt max, and LV ejection fraction at all corresponding stages (Table 3). On day 14 after coronary microembolization, relative to the measurements in the sham group, LV ejection fraction (61%±6% vs 90%±4%, p<0.01), left ventricular systolic pressure (100±10 vs 135±10 mmHg), LV + dP/dt max (2918±276 vs 5707±544 mmHg.s^−1^ and LV - dP/dt max (2481±365 vs 4974±767 mmHg.s^−1^) were significantly lower in HFu rats compared to shams, while LV end diastolic pressure was higher (3±1 vs 18±2 mmHg) (p<0.001 for all). At the end of the study, LV ejection fraction (80%±4% vs 61%±6%), LV + dP/dt max (4828±289 vs 2918±276 mmHg.s^−1^), LV - dP/dt max (4398±269 vs 2481±365 mmHg.s^−1^), and LV systolic pressure (126±13 vs 100±10 mmHg) were significantly higher in HFp rats compared to the HFu group, while LV end diastolic pressure was markedly lower (18±2 vs 13±1 mmHg) (p<0.001 for all).

These data indicate that at the acute phase, LV diastolic and systolic functions are obviously impaired in the untreated rats with HF, and deteriorate continuously during the subacute and chronic phases. In addition, LV functions in PDTC-treated rats are significantly improved at all phases.

## DISCUSSION

HF is associated with multiple adaptations and pathophysiological alterations. These include upgraded systemic vascular resistance, marked LV dysfunction, dilatation, and an activated neuroendocrine system. An understanding of the changes that take place during HF is critical to the study of the disease history and to the efficacy and timing of the interventions that are necessary for reversing the process, or at least retard its progression. Developing an animal model that accurately portrays HF can be of use in HF studies.

Coronary microembolization plays a role in many disorders, including acute coronary syndrome with ST elevation and non-ST elevation, as well as sudden cardiac death. It often occurs after thrombolytic therapy or during coronary interventions (12,13,14,15). It can lead to low or no-reflow during myocardial perfusion, resulting in myocardial infarction and infarct production, coronary reserve reduction, arrhythmias, and contractile dysfunction (16,17,18). Studies have documented that serious coronary microembolization can induce chronic HF (19,20).

Established HF models are evaluated by cardiac function examinations including cardiac catheterization and ultrasonic cardiogram. Based on these evaluations, rats with LV end diastolic pressure >15 mm Hg are thought to have severe congestive HF (21). The myocardial infarction area is correlated well with cardiac function, and when the infarction area surpasses 20%, obvious contractile dysfunction or cardiac shock is induced. Our model mimicked several sequelae of HF such as severely impaired LV diastolic and systolic functions, reduced cardiac function, and LV dilation. These findings indicate that chronic HF characterized by a loss of contractile myocardium can be successfully created using homologous microthrombotic particles. This study’s rat HF approach differs from previous trials in the following manner: previous studies use a single or multiple intracoronary injection of microspheres to induce myocardial dysfunction and the operative procedure is relatively easy (22). However, in the current study, the experimental micro-emboli obtained are abundant in fibrin, platelets, and other particles, which is an attractive feature of the HF model. This model closely mimics aspects of clinical coronary emboli and can be used to simulate clinical CME conditions. The overall area of the infarcts is generally unchanged and can be controlled, as indicated by a preliminary experiment showing dose-dependent but unpredictably distributed microinfarcts.

As a transcription factor sensitive to redox, NF-κB exists in cell types that have the p50/p65 heterodimer. In general, inactive NF-κB dimers are bound to the inhibitor of NF-κB proteins (IκBs) located in the cytosol. Stimuli such as reactive oxygen species (ROS) may activate NF-κB. The activated NF-κB is then translocated into the nucleus, where it activates specific target genes that regulate inflammation and apoptosis. Thus, NF-κB is a major initiator in ROS-mediated cardiomyocyte apoptosis and excessive cardiac fibrosis (23). Accumulating evidence suggests that PDTC, as a powerful antioxidant and inhibitor of NF-κB, can be an ideal therapeutic candidate for many cardiac diseases (24). PDTC protects against adriamycin-induced myocardial apoptosis and reduces ischemia-reperfusion-associated myocardial no-reflow (25,26). Therefore, using our HF rat model, we further studied the effects of PDTC on cardiomyocyte apoptosis and cardiac pathophysiology of HF. The results of the present study are consistent with the evidence that myocyte apoptosis is aggravated if infarcted hearts are left untreated (27,28). The current study further reveals that PDTC treatment can markedly ameliorate myocyte apoptosis in a chronic HF.

In cardiac pathology, the role of cardiac myocyte apoptosis is still speculative. However, it is intriguing that a progressive loss of cardiac myocytes can in part be compensated by fibroblast cell proliferation, which further deteriorates cardiac function. Furthermore, the degree of cardiomyocyte apoptosis is intriguingly associated with the severity of LV dilation and systolic dysfunction in acromegaly-associated cardiomyopathy in humans (29). Other reports show that the shift from concentric LV hypertrophy to the dysfunction of LV dilatation and systolic pressure is linked with aggravated myocyte apoptosis (30,31). Therefore, antioxidant-mediated inhibition of programmed cell death may be beneficial in treating congestive HF.

Since research has shown that oxidative stress has adverse effects on myocardial function (32,33), this study also attempts to probe into the hemodynamic effects of antioxidant therapy. Attenuated oxidative stress within the myocardium probably suppresses the deteriorated myocardial contractility. In our HF model, antioxidant treatment significantly attenuated cardiac dysfunction. Accordingly, we speculate that the apparent benefits of PDTC treatment in HF may be partly due to its antioxidant effect and partly due to the PDTC-mediated transcription inhibition of several pro-apoptotic genes, leading to inhibition of cell death and improved cardiac function. In conclusion, treatment with antioxidant PDTC may be a promising therapeutic candidate for treating congestive HF, but further investigation is needed.

## Figures and Tables

**Table 1:**
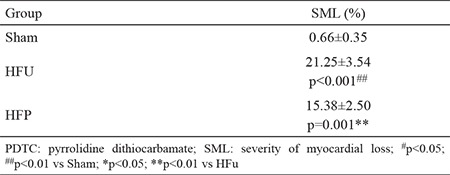
Effects of PDTC on myocardial fibrosis

**Table 2:**
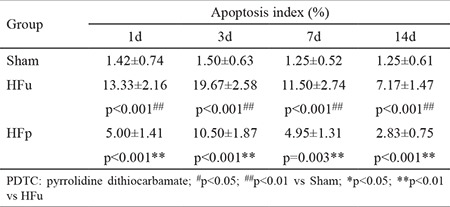
Effects of PDTC on cardiocyte apoptosis at different experimental stages

**Table 3:**
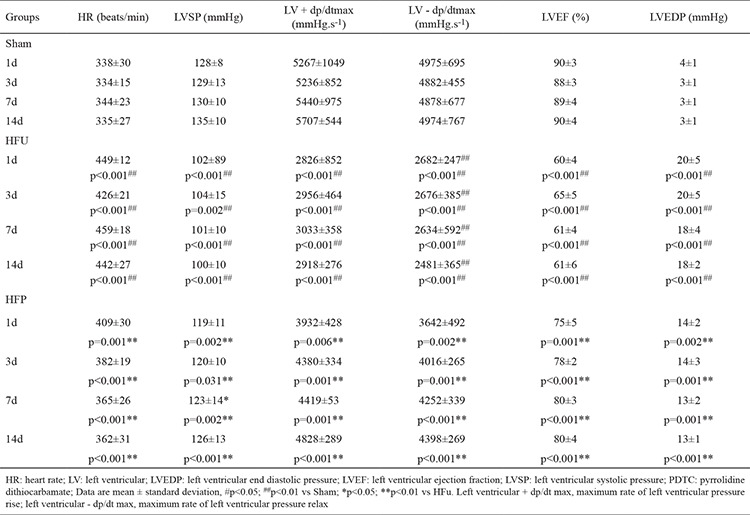
Effects of PDTC on cardiac function assessed using hemodynamics and echocardiography
